# Laparoscopic Omental Lymph Node Flap Transfer for Lower Extremity Lymphedema: Insights into Lymphangiogenesis and Clinical Outcomes

**DOI:** 10.7150/ijms.125568

**Published:** 2026-01-14

**Authors:** Chakrit Eaimkijkarn, Nutthapon Kanasup, Oumyos Rattanamahattana, Peerawan Chochai, Pornthep Sirimahachaiyakul, Worapong Leethochavalit, Tanayos Suyabodha, Chamnong Chirawichada, Wuttichai Saengprakai, Amarit Tansawet, Suphakarn Techapongsatorn

**Affiliations:** 1 Division of Plastic and Reconstructive Surgery, Department of Surgery, Faculty of Medicine, Vajira Hospital, Navamindradhiraj University, Bangkok, Thailand.; 2 Division of Vascular surgery, Department of Surgery, Faculty of Medicine, Vajira Hospital, Navamindradhiraj University, Bangkok, Thailand.; 3 Department of Research and Medical Innovation, Faculty of Medicine, Vajira Hospital, Navamindradhiraj University, Bangkok, Thailand.; 4 Division of General surgery, Department of Surgery, Faculty of Medicine, Vajira Hospital, Navamindradhiraj University, Bangkok, Thailand.

**Keywords:** lymphedema, laparoscopic omental flap, vascularized lymph node transfer, lymphangiogenesis, lower extremity, lymphatic reconstruction

## Abstract

**Background:** Lower extremity lymphedema is a chronic surgical disease marked by lymphatic obstruction, fibrosis, and recurrent infection. Conservative therapy is often inadequate in advanced cases. Vascularized lymph node transfer (VLNT) restores drainage through lymphangiogenesis and node regeneration. The omentum, rich in lymphoid tissue and VEGF-C secretion, is an underutilized donor site. This study evaluated the safety, efficacy, and mechanistic outcomes of laparoscopic omental lymph node flap transfer.

**Methods:** This retrospective case series evaluated, twelve patients (14 limbs) with advanced lower extremity lymphedema underwent laparoscopic omental VLNT. Outcomes included limb circumference reduction, resolution of lymphangitis, lymphoscintigraphic improvement, and donor-site morbidity. Lymphoscintigraphy at 12 months assessed functional restoration and tracer uptake as surrogates of lymphangiogenesis.

**Results:** At a mean follow-up of 27 (12-43) months, the mean circumference reduction was 29%, greatest in distal segments. All recurrent lymphangitis resolved, and chronic wounds healed within 3 months. Lymphoscintigraphy demonstrated enhanced drainage, reduced dermal backflow, and increased tracer uptake within transferred flaps, suggesting functional integration. Flap survival was 93%, with no gastrointestinal complications or donor-site hernia. Patients reported improved skin texture, reduced heaviness, and greater walking tolerance.

**Conclusions:** Laparoscopic omental VLNT is a safe and effective option for refractory lower extremity lymphedema. Clinical improvements were supported by lymphoscintigraphic consistent with lymphangiogenesis and lymphatic restoration. This minimally invasive approach represents an important advancement in physiologic lymphedema surgery.

## Introduction

Lymphedema is a chronic, progressive surgical disease characterized by lymphatic obstruction, protein-rich fluid accumulation, and progressive fibrosis. Clinically, it manifests as limb swelling, recurrent lymphangitis, functional disability, and impaired quality of life 1-3. In high- and middle-income countries, secondary lymphedema most frequently arises from iatrogenic injury, particularly following cancer surgery involving lymphadenectomy or radiotherapy 4. Among patients with gynecologic malignancies, the prevalence of lower limb lymphedema is reported to be as high as 15%, imposing a substantial burden both physically and psychologically 5, 6. The condition negatively impacts mobility, predisposes to recurrent infection, and significantly reduces quality of life and psychosocial health 7-9.

Conservative management, including complete decongestive therapy (CDT), compression garments, and physiotherapy, represents the first-line approach but provides limited benefit in advanced or refractory cases 10. Consequently, surgical procedures have been developed and broadly classified into reductive approaches, aimed at debulking fibrotic tissue, and physiologic approaches, designed to restore or bypass impaired lymphatic pathways. Among physiologic techniques, lymphaticovenular anastomosis (LVA) has demonstrated effectiveness in early-stage disease, whereas vascularized lymph node transfer (VLNT) is generally preferred for advanced lymphedema 11-13.

VLNT has shown favourable outcomes, including limb volume reduction, decreased lymphangitis episodes, and functional improvement 14-16. Several donor sites have been described, including submental 14, supraclavicular 15, axillary 16, groin 17, and omental lymph nodes 18. Each site has distinct advantages and drawbacks. Importantly, harvesting from the groin or axilla may result in donor-site lymphedema, prompting the search for safer alternatives 19, 20.

The omentum offers unique advantages as a donor site: it is rich in lymphoid aggregates (milky spots), secretes vascular endothelial growth factor-C (VEGF-C) to promote lymphangiogenesis 21, and possesses intrinsic lymphovenous communications that facilitate direct drainage into the systemic circulation. Furthermore, laparoscopic harvest minimises donor-site morbidity and reduces the risk of abdominal complications 18, 22, 23. Early clinical reports have shown encouraging results with omental VLNT, but the biological mechanisms underlying its efficacy in lower extremity lymphedema remain underexplored.

This study aimed to assess the clinical efficacy of laparoscopic omental lymph node flap transfer for advanced lower extremity lymphedema, while exploring lymphoscintigraphic findings suggestive of lymphangiogenesis and *de novo* lymph node formation.

## Materials and Methods

### Study design and ethical approval

We conducted a retrospective case series (Level of Evidence IV) involving consecutive patients with lower extremity lymphedema who underwent laparoscopic omental flap transfer between July 2016 and February 2019 at Vajira Hospital, Bangkok, Thailand. The study protocol was approved by the Institutional Review Board of the Faculty of Medicine, Vajira Hospital (COA139/2019) and complied with the Declaration of Helsinki. Written informed consent to publish images was obtained.

### Patient selection

Patients eligible for this procedure had advanced-stage lower extremity lymphedema (Campisi stages II-IV) and had previously undergone conservative treatments that produced unsatisfactory results. Each patient was evaluated preoperatively for limb circumference, symptoms, and lymphoscintigraphic findings. Of the 12 patients included, seven reported recurrent lymphangitis, and two had undergone prior lymphaticovenular anastomosis (LVA) with limited success. The duration of symptoms averaged 6 ± 5 years.

### Surgical technique

**Figure 1:**
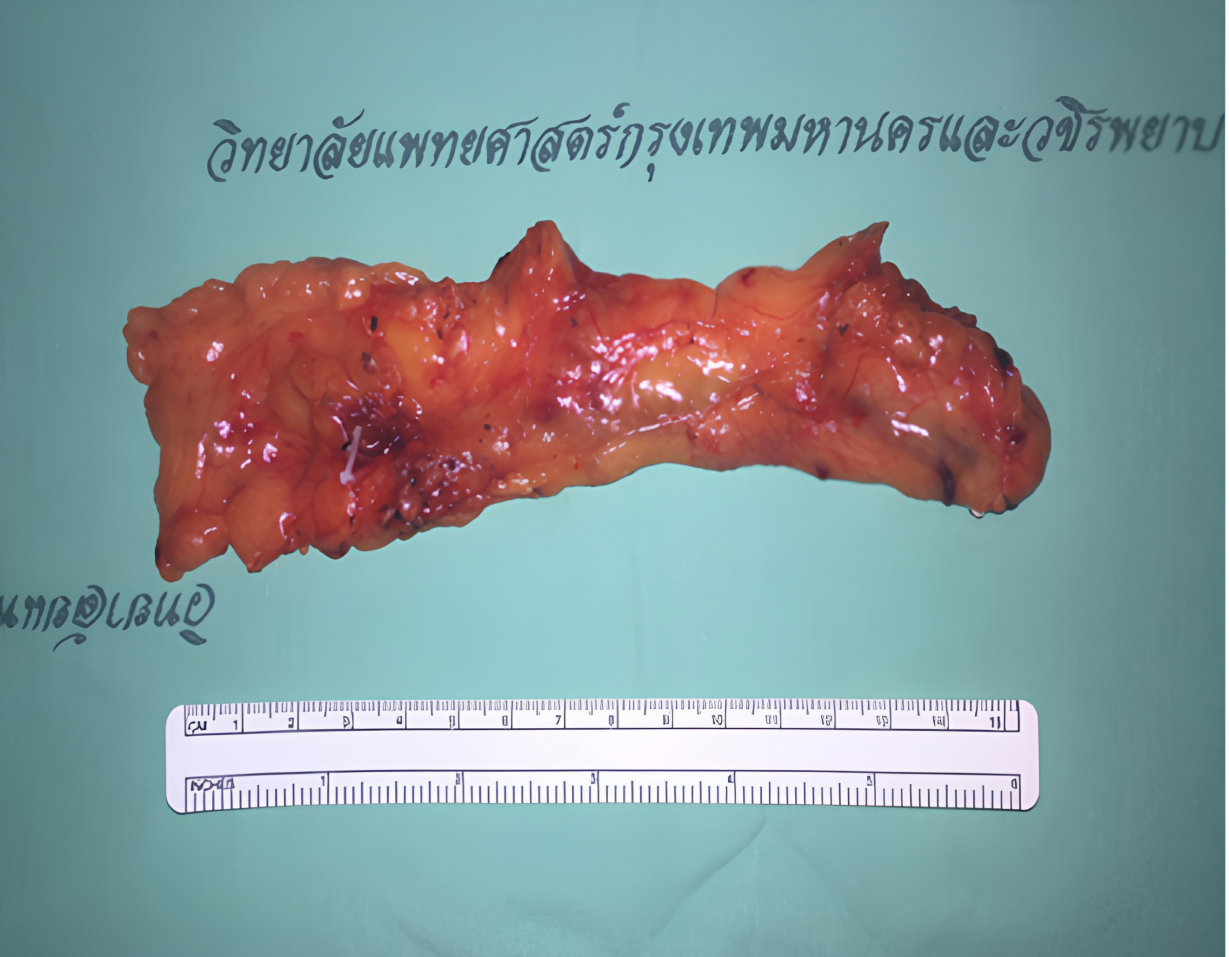
Omental flap based on the right gastroepiploic vessels.

A two-team approach was employed. Under general anaesthesia, the omentum was harvested laparoscopically using four abdominal ports. Dissection was initiated along the greater curvature of the stomach to mobilize the omentum, with preservation of the left gastroepiploic vessels. The right gastroepiploic artery and vein were ligated at their origin to isolate the pedicle. Care was taken to preserve lymph node-bearing tissue within the omental flap (Figure 1).

**Figure 2:**
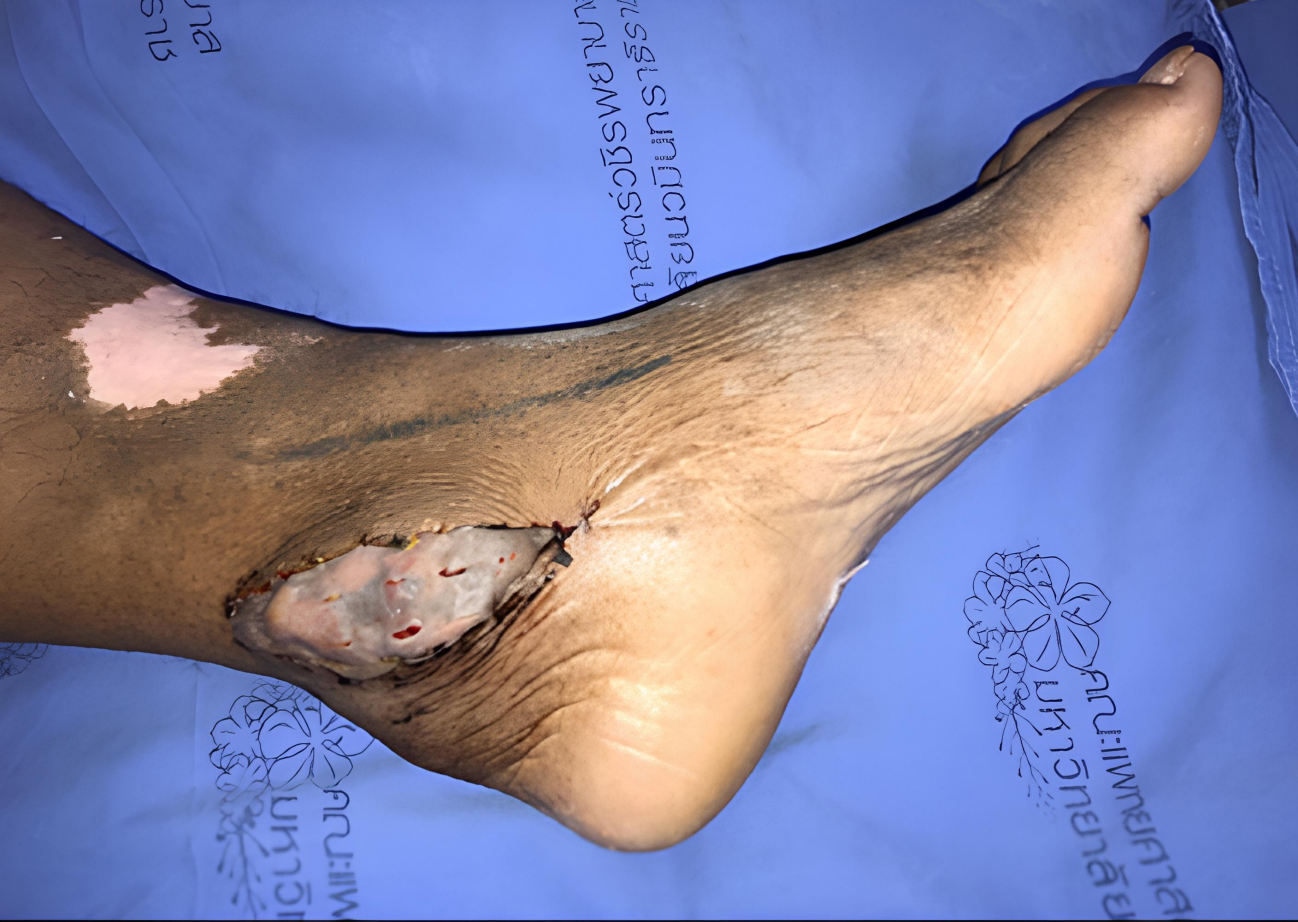
Omental flap with skin graft coverage at the ankle of the lymphedematous limb.

The ankle was selected as the recipient site to optimize gravitational assistance in lymphatic drainage. Microvascular anastomosis was performed between the flap pedicle and either the posterior tibial or dorsalis pedis artery and vein. A split-thickness skin graft was applied to cover the flap and surrounding soft tissue (Figure 2).

### Postoperative care

Patients were monitored postoperatively for flap viability, vascular compromise, and systemic complications. Oral feeding resumed on postoperative day 1, and ambulation was encouraged by day 7. CDT was reinstituted one month after surgery to support lymphatic function.

### Outcome measures

Outcomes included limb circumference reduction, resolution of lymphangitis, lymphoscintigraphic improvement, and donor-site morbidity. Donor-site morbidity was assessed through physical examination during follow-up visits to detect hernia or abdominal wall weakness.^24^, ^25^ The circumference reduction rate was calculated using the following formula:

Reduction rate (%) = 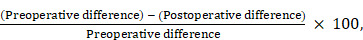


where "Difference" is defined as the affected limb circumference (AL) minus the healthy limb circumference (HL).

For bilateral cases, the absolute reduction was calculated by subtracting postoperative from preoperative measurements at fixed anatomical points. Secondary outcomes included patient-reported changes in skin texture, pain, discomfort, and activity limitation. Lymphoscintigraphy was repeated at 12 months postoperatively to evaluate functional lymphatic restoration.

### Statistical analysis

Descriptive statistics were used to summarise patient demographics, surgical details, and clinical outcomes. Continuous variables were reported as means ± standard deviations or medians with interquartile ranges, depending on distribution. No formal hypothesis testing or inferential statistics were performed due to the small sample size and the exploratory nature of this investigation.

## Results

### Operative and perioperative outcomes

Patient demographics and surgical characteristics are detailed in Table **1.** The cohort included **12** patients with a mean age of **54.7** ± **10.6** years (median: **55.0**; range: **38**-**70)** and a mean body mass index (BMI) of **29.2** ± **4.9** kg/m**² (**median: **28.7**; range: **23.7**-**40.1).** The duration of lymphedema symptoms prior to surgery averaged **63.0** ± **40.3** months (median: **60.0**; range: **12**-**132).** Regarding surgical metrics, the mean total operative time was **324.2** ± **70.1** minutes (median: **352.5**; range: **170**-**420).** The harvested omental flaps had an average size of **26.6** ± **7.5** cm**² (**median: **24.8**; range: **16**-**40)** and a mean weight of **17.6** ± **8.9** g (median: **14.0**; range: **7**-**34).** Postoperative outcomes demonstrated a low mean pain score on day **1** of **2.1** ± **0.7** (median: **2.0**; range: **1**-**3).** The mean length of hospital stay was **15.9** ± **5.5** days (median: **15.5**; range: **7**-**24)**, and patients were followed for a mean duration of **26.8** ±** 10.4** months (median: **28.0**; range: **12**-**43).** Thirteen of 14 flaps (93%) survived without major complications. One flap was lost due to severe venous congestion, later attributed to unrecognised iliac vein stenosis consistent with May-Thurner syndrome. Two additional flaps required surgical revision for venous outflow obstruction. Partial necrosis of the split-thickness skin graft was observed in five patients and was successfully managed with secondary grafting. Crucially, no complications specifically related to the abdominal harvest—such as bowel perforation, visceral injury, hemorrhage, or surgical site infection (SSI)—were encountered in our series.

### Circumference reduction

At a median follow-up of 28 months, the average reduction in limb circumference among unilateral lymphedema cases was 29%. Segmental analysis demonstrated the following reductions:

- 10 cm above the patella: 26%
- Upper patellar border: 26%
- 10 cm below the patella: 31%
- Ankle: 29%
- Midfoot: 32%

**Figure 3:**
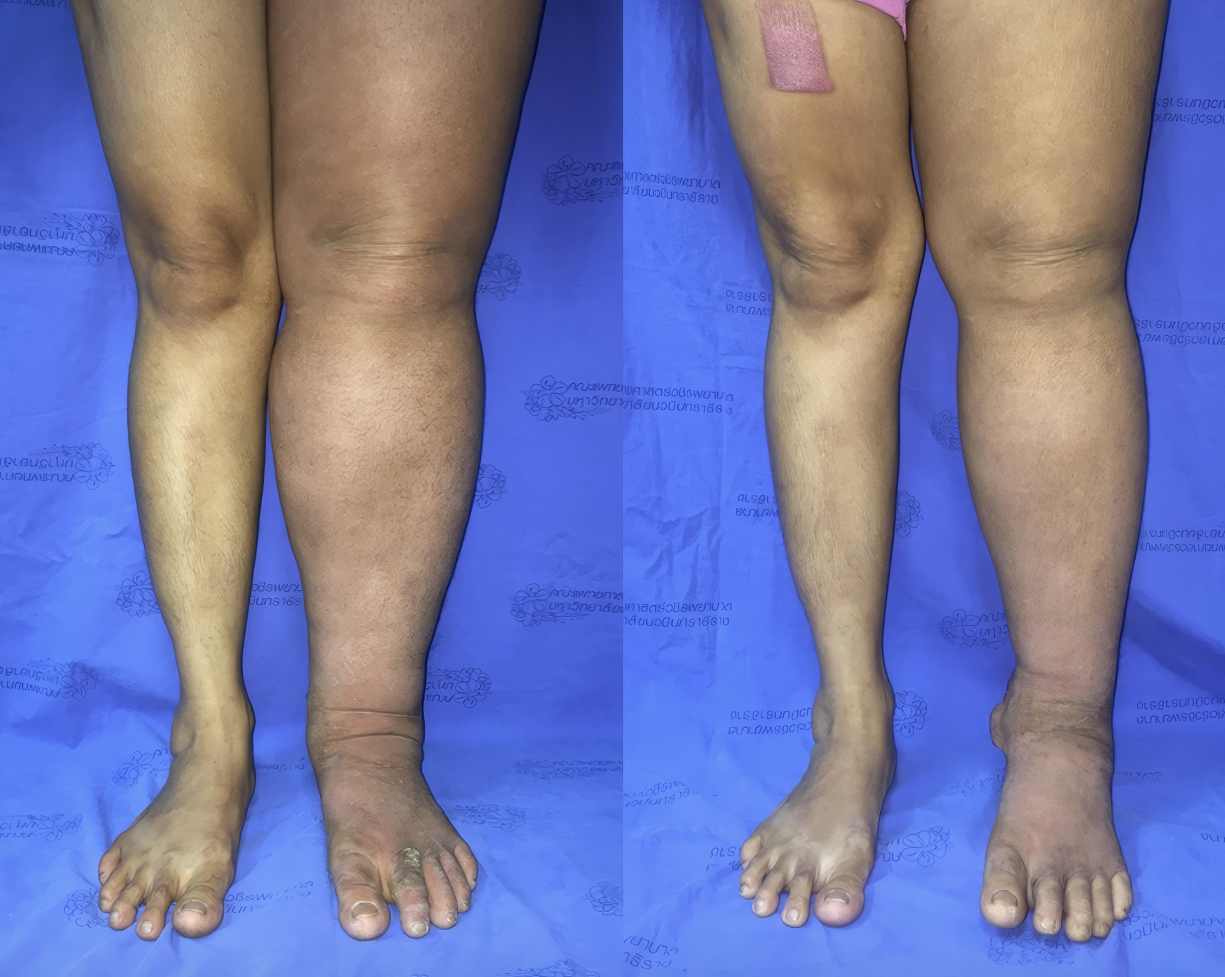
(A) Preoperative photograph of the left leg with lymphedema. (B) Postoperative photograph taken at the 12-month follow-up after omental transfer to the ankle. The mean reduction in limb circumference was 26%.

**Figure 4:**
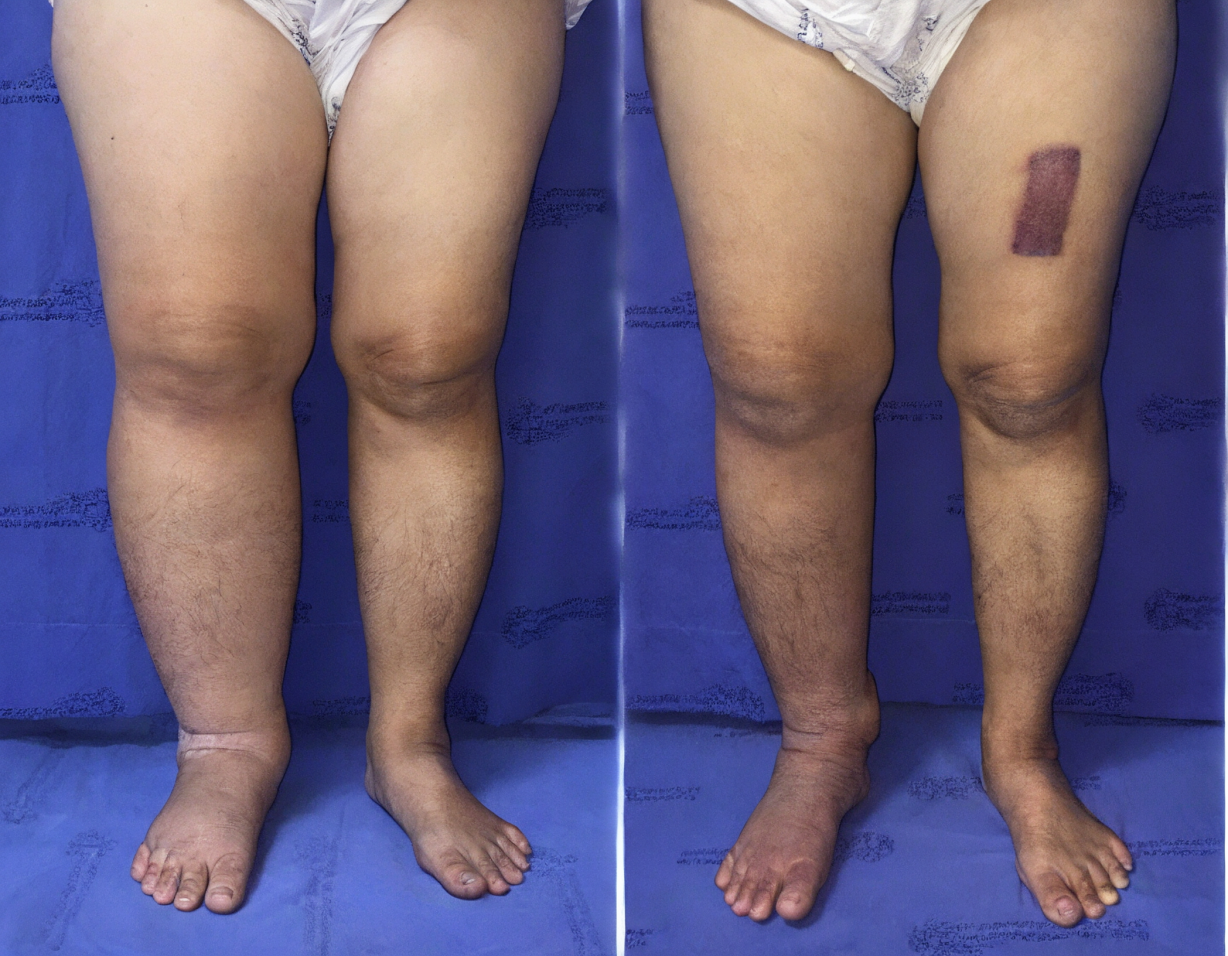
(A) Preoperative photograph of the right leg with lymphedema. (B) Postoperative photograph taken at the 12-month follow-up after omental transfer to the ankle. The mean reduction in limb circumference was 37%.

In patients with bilateral disease (n = 2), the mean absolute reduction across measured levels was 2.4 cm. Details are presented in Table 2, with representative cases shown in Figures 3 and 4; Table 3.

### Clinical outcomes

All seven patients with a preoperative history of recurrent lymphangitis experienced complete resolution postoperatively. Three patients with chronic wounds achieved complete healing within 3 months. Subjective reports included reduced limb heaviness and tightness, improved skin texture and pliability, and increased tolerance for walking and prolonged standing.

### Lymphoscintigraphy findings

**Figure 5:**
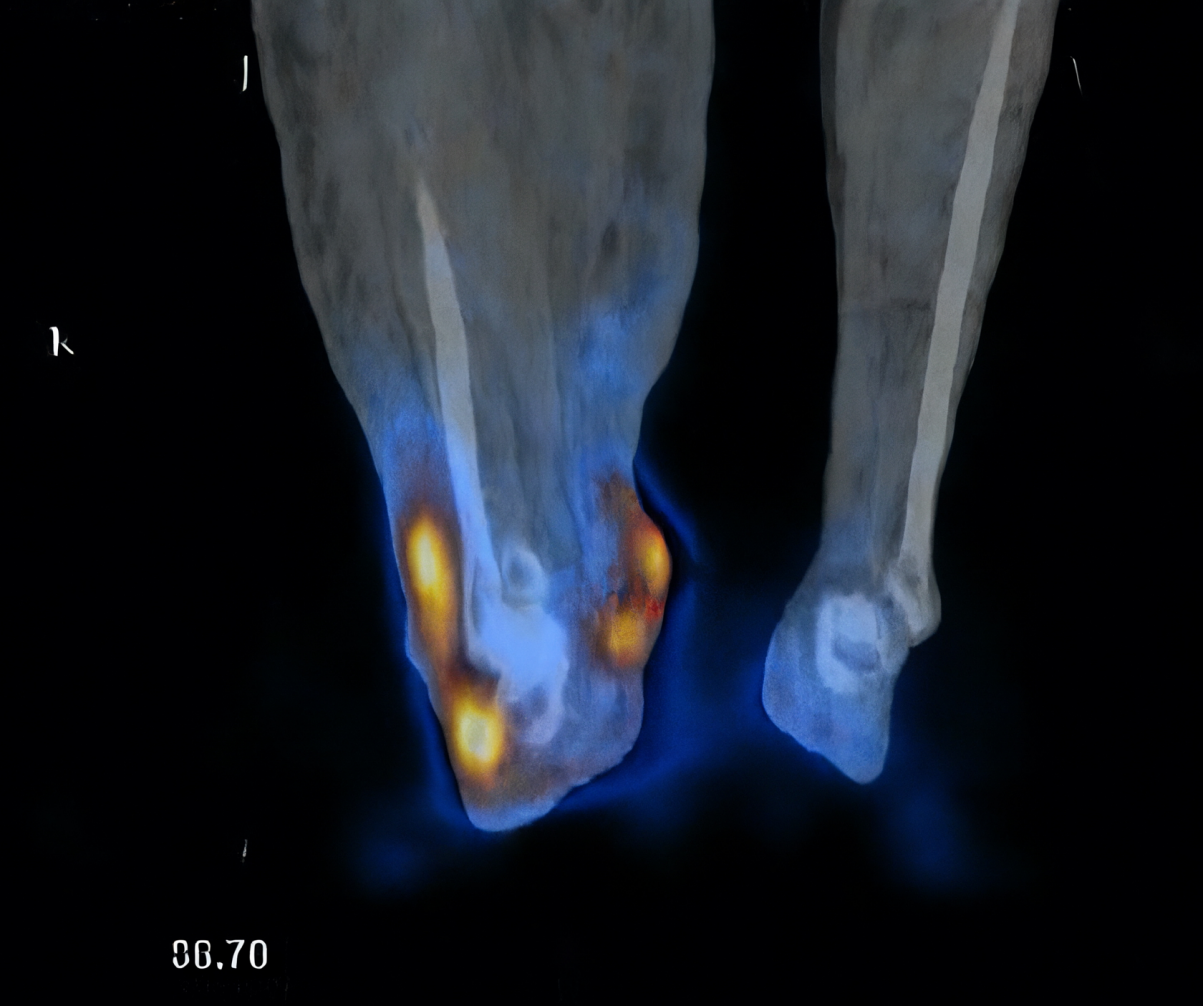
Radiotracer uptake detected in the omental flap (indicated by red dots).

**Figure 6:**
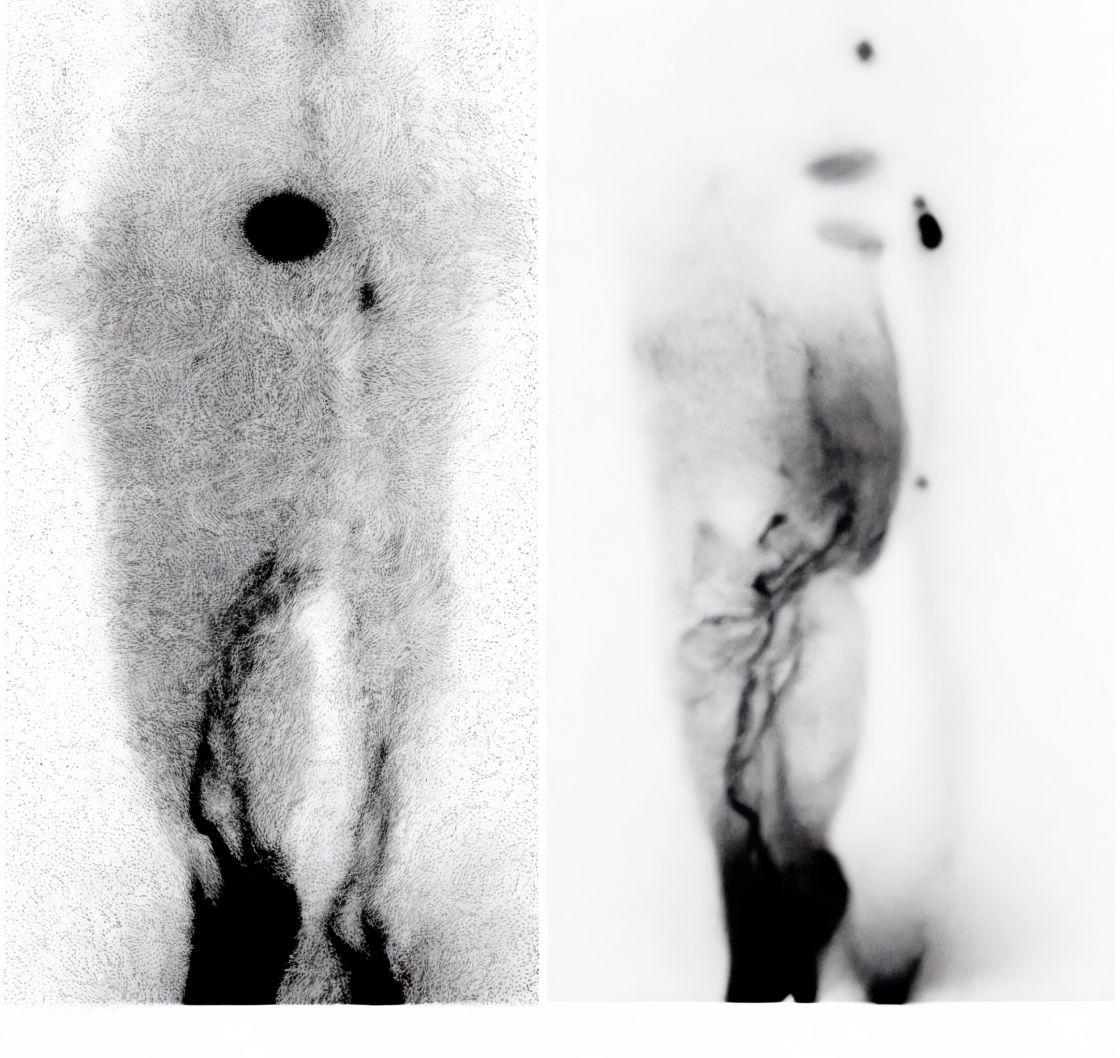
(A) Preoperative lymphoscintigraphy showing dermal backflow throughout the right leg, with main lymphatic vessels visible only below knee level. (B) Lymphoscintigraphy one year postoperatively showing significant improvement in lymphatic drainage. Main lymphatic vessels were visible above knee level, with reduced dermal backflow.

Seven patients underwent lymphoscintigraphy at 12 months. All demonstrated findings suggestive of functional improvement, including increased tracer uptake in the transferred omental flap (Figure 5), clearer visualisation of lymphatic trunks, reduced dermal backflow, and fewer collateral channels (Figure 6). Comparative analysis was not feasible in five patients due to flap failure (n = 1), loss to follow-up (n = 1), or incompatible preoperative imaging protocols (n = 3).

## Discussion

This study demonstrated that laparoscopically harvested omental lymph node flap transfer is a safe and effective surgical option for patients with advanced-stage lower extremity lymphedema. The procedure yielded a mean limb circumference reduction of 29%, complete resolution of lymphangitis, improved lymphoscintigraphic findings, and favorable patient-reported outcomes, including improved skin quality and increased ambulatory function. The flap survival rate was 93%, with no reported donor-site hernia, gastrointestinal complications, or new-onset morbidity during the follow-up period.

These findings are consistent with previous reports by Ciudad et al.^12^ and Nguyen et al. 18, 22, who demonstrated clinical and functional improvement following laparoscopic omental VLNT. Our results are further corroborated by recent extensive clinical experiences reported by Pozzi et al., which reaffirmed that gastroepiploic VLNT is a highly effective and safe procedure for extremity lymphedema, offering significant improvements in limb circumference and tissue tonicity with minimal donor-site morbidity 26. Notably, our lymphoscintigraphy suggested not only improved drainage but also radiotracer uptake within the transferred flap, supporting the concept of neo-lymph node formation through lymphangiogenesis and functional integration into the host lymphatic network. This aligns with prior experimental studies showing that transplanted lymph nodes secrete VEGF-C and VEGF-D, which stimulate VEGFR-3-mediated lymphangiogenesis and promote the regeneration of lymphatic sinuses and high endothelial venules (HEVs) 12, 18, 22.

Compared with conventional donor sites such as the submental 14, supraclavicular 15, and groin lymph nodes 17, the omentum provides unique mechanistic advantages. It harbors abundant lymphoid aggregates (“milky spots”), known to function as niches for neo-lymph node formation, and demonstrates strong VEGF-C activity to accelerate lymphangiogenesis 23. Moreover, its intrinsic lymphovenous connections facilitate direct shunting of lymphatic fluid into the venous system, enhancing early functional integration of the flap 22. These biological features explain why our cohort demonstrated not only clinical improvement but also imaging evidence consistent with de novo lymph node regeneration 16, 20, 27.

The minimally invasive laparoscopic approach also conferred perioperative advantages, including reduced pain, low risk of adhesions and incisional hernia, and favourable cosmetic results. Our flap survival rate of 93% was comparable to prior series, highlighting the reliability of this technique. Importantly, the one case of flap failure due to May-Thurner syndrome underscores the need for careful preoperative venous evaluation, since impaired venous return may compromise both flap perfusion and subsequent lymphangiogenesis.

The observed greater reduction in distal limb circumference further supports the biological mechanism: placing the omental flap at the ankle optimises gravitational drainage and provides a local microenvironment for lymphangiogenesis. The improved uptake and reduced dermal backflow on lymphoscintigraphy are consistent with the establishment of new lymphatic pathways and the functional maturation of neo-lymph nodes.

Skin graft necrosis, observed in five patients, remains a technical challenge. Future refinements—such as negative pressure wound therapy or flap anchoring modifications—may help reduce this complication.

The strengths of this study include a relatively long follow-up duration (mean 26.8 + 10.4 months; median: 28.0; range: 12-43), the application of a consistent microsurgical technique by a specialised team, and the incorporation of both clinical and imaging endpoints. However, several limitations must be acknowledged. These include the retrospective design, the small sample size, and lack of validated quality-of-life instruments to quantify patient-reported outcomes. Furthermore, postoperative lymphoscintigraphy was not available for comparison in five cases due to flap failure, loss to follow-up, or incompatible preoperative imaging protocols. Finally, no histologic or molecular analysis was performed to definitively confirm lymphangiogenesis; thus, our mechanistic conclusions rely solely on indirect functional imaging.

Future research should focus on multicenter prospective studies comparing omental VLNT with other donor sites, incorporating validated patient-reported outcome measures, and using high-resolution modalities such as indocyanine green (ICG) lymphography. In addition, mechanistic studies should investigate molecular markers of neo-lymph node development, confirming whether omental flaps function as true lymphoid organs capable of long-term immune and lymphatic restoration.

Furthermore, the integration of surgical interventions with conservative therapies remains a cornerstone of effective lymphedema management. As highlighted by Farid et al. 27, a comprehensive treatment algorithm that incorporates VLNT can yield substantial functional improvements even in challenging cases, reinforcing the importance of a multimodal approach to achieve optimal long-term outcomes.

In conclusion, laparoscopic omental lymph node flap transfer achieves substantial improvements in limb volume, lymphatic function, and patient comfort, with minimal donor-site morbidity. Beyond clinical efficacy, our results highlight mechanistic findings supportive of lymphangiogenesis and de novo lymph node formation, reinforcing the role of the omentum as both a safe donor site and a biologically active substrate for lymphatic regeneration.

## Conclusion

Laparoscopy-assisted omental flap transfer serves as a safe and minimally invasive option for treating advanced-stage lower extremity lymphedema. Our findings demonstrate substantial reductions in limb circumference, restoration of lymphatic drainage, and favorable lymphoscintigraphic findings. Notably, the increased radiotracer uptake within the transferred flaps suggests functional integration and provides finding consistent with lymphangiogenesis. The use of the omentum as a donor source offers key advantages, including abundant lymphoid tissue and intrinsic lymphovenous connections, while the laparoscopic approach minimizes surgical trauma and donor-site morbidity. Future research utilizing larger, multicenter cohorts is warranted to further validate these clinical and functional outcomes.

## Figures and Tables

**Table 1:** Clinical and surgical data of patients undergoing laparoscopic omental lymph node flap transfer.

Patient No.	Patient Age (years)	BMI	Diagnosis	Previous Surgery	Affected Limb	Campisi Stage	Duration of Symptoms (months)	Previous LVA	Flap Size (cm²)	Flap Weight (grams)	Total Operative Time (min)	Pain Scale Postoperative Day 1	Donor Site Complication	Recipient Site Complication	Additional Surgery	Length of Stay (days)	Follow-Up (months)
1	70	23.7	Cervical cancer	TAH+BSO	Left leg	4	60	Yes	4x7	17	360	2	None	Partial STSG necrosis	STSG	24	43
2	50	30.1	Cervical cancer	TAH+BSO+GND	Left leg	4	72	Yes	4x8	23	360	2	None	None	None	14	40
3	38	27.2	Cervical cancer	TAH+PND	Left leg	4	12	No	3x7	12	390	3	None	Partial STSG necrosis	STSG	16	37
4	65	24.0	Cervical cancer	TAH+BSO	Left leg	2	72	No	4x8	30	345	2	None	Partial STSG necrosis	None	16	32
5	53	25.2	Cervical cancer	TAH+PND	Left leg	2	60	No	3x7	34	260	1	None	Flap ischemia (venous obstruction)	Flap removal	8	31
6	40	34.0	Primary lymphedema	None	Both legs	2	120	No	R 2.5x9 L 2.5x9	R 8 L 7	360	2	None	Partial STSG necrosis	STSG	15	29
7	49	32.7	Endometrial cancer	TAH+BSO+PND	Right leg	3	24	No	3x9	10	285	2	None	None	None	22	27
8	65	30.7	Endometrial cancer	TAH+PND	Right leg	4	120	No	4x10	20	250	2	None	None	None	22	22
9	66	30.7	Endometrial cancer	TAH+BSO+PND	Both legs	3	48	No	R 3x7 L 3x7	R 14 L 14	420	2	None	Flap ischemia (venous obstruction)	Flap revision	21	19
10	45	40.1	Trauma	Debridement at left leg	Left leg	3	36	No	5x8	34	360	1	None	Partial STSG necrosis	STSG	13	17
11	58	25.0	Endometrial cancer	TAH+BSO+PND, Left iliac vein stent	Left leg	3	108	No	2x8	10	170	3	None	None	None	7	13
12	57	27.0	Necrotizing fasciitis	Debridement at left leg	Left leg	4	132	No	4x7	14	330	3	None	Partial STSG necrosis	STSG	13	12

Notes: BMI = body mass index; TAH = total abdominal hysterectomy; BSO = bilateral salpingo-oophorectomy; PND = pelvic node dissection; GND = groin node dissection; STSG = split-thickness skin graft; LVA = lymphaticovenular anastomosis.

**Table 2:** Percentage reduction in limb circumference at each anatomical level in patients with unilateral lymphedema.

Patient No.	Circumference Reduction Above Knee (%)	Circumference Reduction Knee (%)	Circumference Reduction Below Knee (%)	Circumference Reduction Ankle (%)	Circumference Reduction Foot (%)	Circumference Reduction Mean (%)
1	17	22	22	42	25	26
2	12	38	15	23	8	19
3	36	53	32	10	0	26
4	14	44	42	38	50	38
7	47	35	44	17	40	37
8	8	4	15	13	31	14
10	67	20	60	64	67	56
11	14	14	0	23	0	10
12	20	0	50	29	67	33
Average	26	26	31	29	32	29

Data are presented as percentage reduction at five levels: above knee, knee, below knee, ankle, and foot.

**Table 3:** Absolute reduction in limb circumference (in cm) at each anatomical level in patients with bilateral lymphedema.

Patient No.	Circumference Reduction Above Knee (cm)	Circumference Reduction Knee (cm)	Circumference Reduction Below Knee (cm)	Circumference Reduction Ankle (cm)	Circumference Reduction Foot (cm)	Circumference Reduction Mean (cm)
	Right	Left	Right	Left	Right	Left
6	0	2.5	8.5	8	4	3.5
9	4.5	5.5	3.5	4	0	0
Average	2.25	4	6	6	2	1.75
